# Cavernous Hemangioma in the Parotid Gland of an Adult: A Case Report and Review of Literature

**DOI:** 10.7759/cureus.52285

**Published:** 2024-01-15

**Authors:** Farhan M Alanazi, Saeed Alqahtani, Sultan H Alruwaili, Abdullah A Alzamil, Fareed R AlGhamdi

**Affiliations:** 1 Otolaryngology-Head and Neck Surgery, Prince Sultan Military Medical City, Riyadh, SAU; 2 Otolaryngology-Head and Neck Surgery, Prince Mohammed Medical City, Jouf, SAU; 3 Medicine, Jouf College of Medicine, Jouf, SAU; 4 Medicine and Surgery, Unaizah College of Medicine, Unaizah, SAU

**Keywords:** mass, vascular tumors, parotid gland, salivary glands, hemangiomas

## Abstract

Hemangiomas are benign vascular tumors and are classified into cavernous, capillary, and mixed, with the head and neck area as the most common site. Hemangiomas are common in pediatrics and rare in adults. Diagnosing cavernous hemangioma is challenging and requires a complete history, proper physical examination, and several radiological modalities to improve diagnostic accuracy because it is uncommon in adults. Herein, we present a case of a 66-year-old female Saudi patient with cavernous hemangioma from the diagnosis until the surgical treatment. No previous studies are reported in Saudi Arabia and this is a rare presentation of cavernous hemangioma at this age. Cavernous hemangioma in the parotid gland in adults is uncommon and is difficult to diagnose. Therefore, a thorough physical examination and several radiological modalities are required to improve diagnostic accuracy. The most effective treatment of cavernous hemangioma in adults is surgical resection.

## Introduction

Hemangiomas are benign vascular tumors caused by increased endothelial cell proliferation and turnover [1] and are categorized into cavernous, capillary, and mixed. Hemangiomas can occur anywhere on the body, with 65% of cases starting in the head and neck areas. The most commonly impacted structure is the salivary glands, with the parotid gland accounting for 80%-85% of all cases [2-4]. However, adult hemangiomas in the parotid gland only comprise 0.4%-0.6% of the total parotid gland tumors [2-4]. Typically, parotid hemangioma affects children under the age of one year. It comprises 50% of all parotid gland tumors and is characterized by rapid development and progression between the ages of six and eight months, followed by an involution period in the first decade [3,5]. Conversely, parotid hemangioma is uncommon in adults and does not regress [3,4,6].

## Case presentation

**Figure 1:**
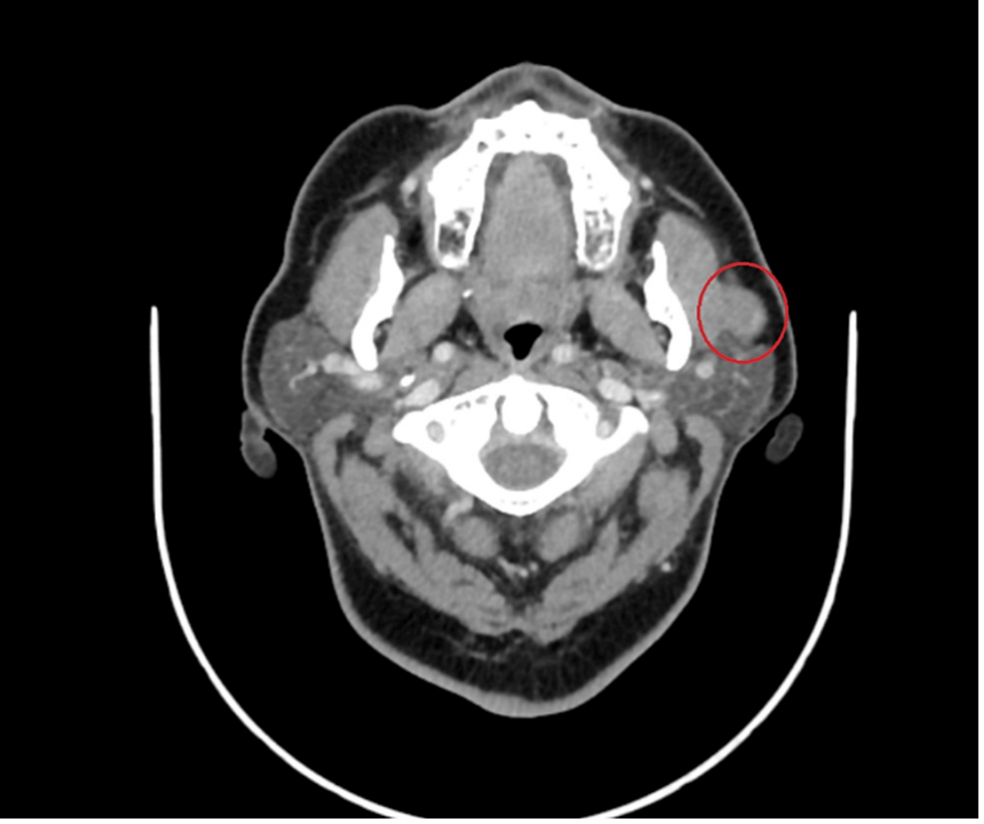
CT head and neck (axial section) An enlarged, non-enhanced oval shaped left mandibular mass located superficial to and lying on the left masseter muscle measuring 1.2 cm with foci of calcification; minimal peripheral enhancing components.

**Figure 2:**
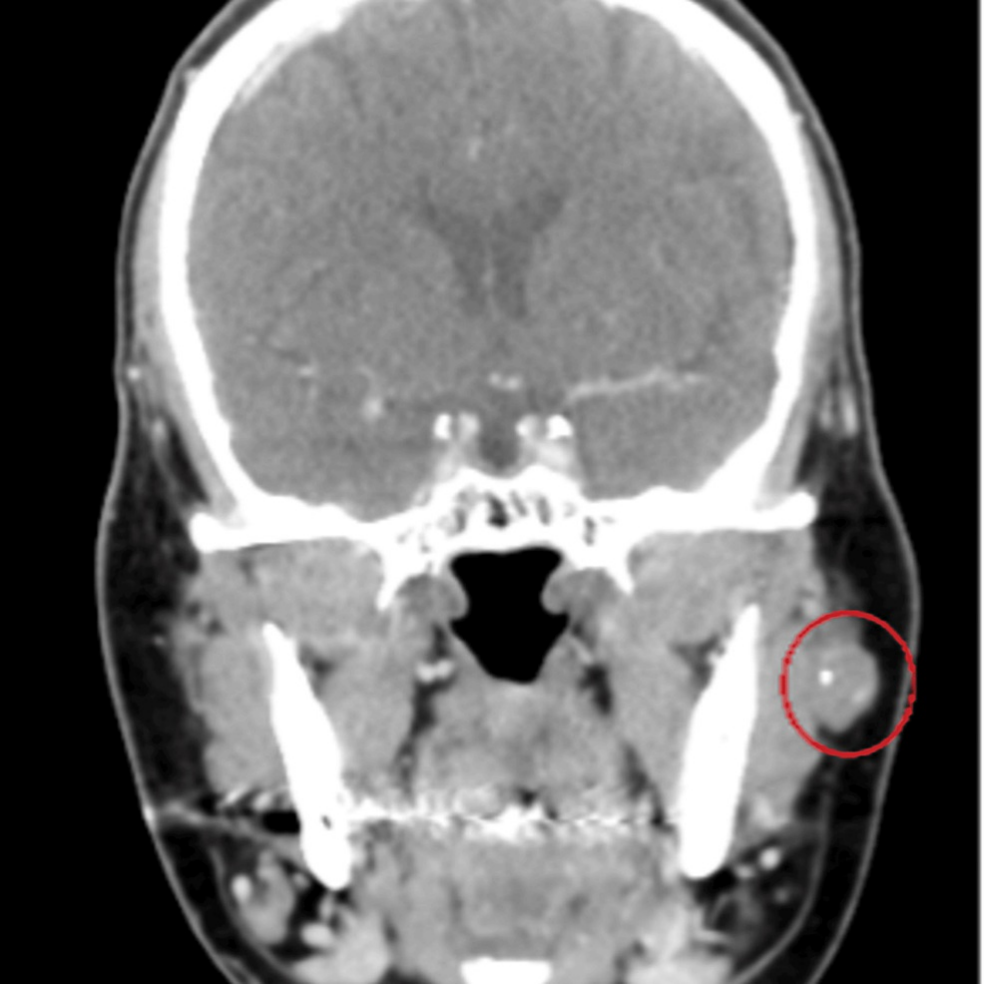
CT head and neck (coronal section) An enlarged, non-enhanced oval shaped left mandibular mass located superficial to and lying on the left masseter muscle measuring 1.2 cm with foci of calcification; minimal peripheral enhancing components.

A 66-year-old female with diabetes mellitus and hypertension presented in our clinic complaining of left pre-auricular swelling for >one year in a stable size. She denied any history of pain, numbness, or facial weakness, as well as night sweating, fever, or weight loss. Local examination revealed a non-fixed pre-auricular mass, 1 × 1 cm in size, with firm, non-tender, and well-defined borders. Head and neck examination revealed no palpable lymph nodes and facial nerves were bilaterally intact. Flexible nasal endoscopy revealed bilateral hypertrophied turbinate, clear nasopharyngeal cavity with no masses, and clear bilateral mobile vocal cords. The parotid gland ultrasonography revealed a focal-shaped hypoechoic well-demarcated lesion within the internal focus of calcification seen superficial to the left masseter muscle closely anterior to the left parotid but looks extra-parotid, measuring 1.7 × 0.8 cm. Head and neck contrast-enhanced computed tomography (CT) revealed an enlarged, non-enhanced oval shape left mandibular mass located superficial to and lying on the left masseter muscle measuring 1.2 cm with foci of calcification; minimal peripheral enhancing components; bilaterally enlarged lymph nodes at IB and IIB levels with the largest preserved fatty hilum on the left IB level measuring 1.1 cm (Figures 1, 2).

The patient underwent left superficial parotidectomy and intraoperative frozen section which revealed a cavernous hemangioma without evidence of malignancy. The patient was doing fine postoperatively, with no active complaint, the facial nerve was intact, and the rest of the examination is unremarkable. The patient was followed in the clinic until the wound was healed.

## Discussion

The World Health Organization Classification of Tumors, established in 2005, reported 50 cases of adult hemangiomas globally, highlighting the disorder’s rarity [1,7,8]. Cavernous hemangioma is the only parotid hemangioma reported in the literature, with no current data on its incidence in the adult population [9]. Additionally, a recent study by Nagao et al. published a case series of 20 cases of cavernous hemangioma in adult patients and revealed 26 years of age as the mean [10]. Cavernous hemangioma of the parotid gland in adults must be reported because of this unusual discovery.

Cavernous hemangioma of the parotid gland frequently appears as a mass in the parotid region, along with a red-blue macule or papule that may or may not be present and a pulsating sensation when the mass is palpated. However, the case is solely presented as a mass in adults with no apparent relationships. For instance, the usual clinical presentation of patients in multiple case studies was a slowly expanding and asymptomatic mass over the parotid region with no apparent relationships [1,3,11,12].

Salivary gland tumors can be diagnosed using many imaging techniques. An important initial tool is an ultrasound [6,13]. Ultrasound reveals a mass with well-defined borders and either homogenous or heterogeneous echogenicity in cavernous hemangioma cases [14]. However, the sonographic results are not typical. Fine needle aspiration is performed preoperatively in parotid masses, although it is contraindicated in vascular lesions because iatrogenic hematoma may occur [3,6,15]. Magnetic resonance imaging assesses the parotid hemangioma’s extension [16,17]. Hemangiomas typically manifest as a well-defined, lobulated, and uniformly enhancing lesion. The lesion is uniformly hypointense on the T1-weighted sequence but hyperintense with varying vascularity on the T2-weighted sequence. Cavernous hemangiomas of the parotid gland are rarely diagnosed with angiography although this technique is the gold standard for identifying arteriovenous malformations [14,18]. Cavernous hemangioma of the parotid is a rare and unusual tumor that develops in adulthood, making its diagnosis challenging just through clinical examination and imaging methods [6,19].

Adult parotid gland hemangiomas do not regress, thus surgical intervention is required in contrast to infantile hemangiomas that could involute or be treated non-surgically with sclerotherapy, propranolol, intralesional, or systemic corticosteroids [6,20,21]. Preoperative embolization is important for minimizing the size of large hemangiomas, as well as intraoperative bleeding [20]. Non-surgical therapies are only considered in adults if surgery is not an option.

## Conclusions

Cavernous hemangioma in the parotid gland is uncommon in adults. It is difficult to diagnose, thereby necessitating a thorough physical examination and several radiological modalities to improve diagnostic accuracy. This article presents the case of a 66-year-old female Saudi patient with cavernous hemangioma from the diagnosis until the surgical treatment. Nowadays, surgical resection is the most effective interventional treatment for cavernous hemangioma in the parotid gland.
